# Genetic Markers for Later Remission in Response to Early Improvement of Antidepressants

**DOI:** 10.3390/ijms21144884

**Published:** 2020-07-10

**Authors:** Hee-Ju Kang, Ki-Tae Kim, Kyung-Hun Yoo, Yoomi Park, Ju-Wan Kim, Sung-Wan Kim, Il-Seon Shin, Ju Han Kim, Jae-Min Kim

**Affiliations:** 1 Department of Psychiatry, Chonnam National University Medical School, Gwangju 61469, Korea; hjkang82@chonnam.ac.kr (H.-J.K.); tarot383@naver.com (J.-W.K.); swkim@chonnam.ac.kr (S.-W.K.); sshin@chonnam.ac.kr (I.-S.S.); 2 Department of Laboratory Medicine, Korea University Anam Hospital, Seoul 02841, Korea; kitae@snu.ac.kr; 3 Seoul National University Biomedical Informatics (SNUBI), Division of Biomedical Informatics, Seoul National University College of Medicine, Seoul 151-742, Korea; yookyunghun@gmail.com (K.-H.Y.); yoomip89@gmail.com (Y.P.)

**Keywords:** antidepressant, treatment outcome, remission, improvement, genetic marker, whole-exome sequencing

## Abstract

Planning subsequent treatment strategies based on early responses rather than waiting for delayed antidepressant action can be helpful. We identified genetic markers for later non-remission in patients exhibiting poor early improvement using whole-exome sequencing data of depressive patients treated in a naturalistic manner. Among 1000 patients, early improvement at 2 weeks (reduction in Hamilton Depression Rating Scale [HAM-D] score ≥ 20%) and remission at 12 weeks (HAM-D score ≤ 7) were evaluated. Gene- and variant-level analyses were conducted to compare patients who did not exhibit early improvement and did not eventually achieve remission (*n* = 126) with those who exhibited early improvement and achieved remission (*n* = 385). Genes predicting final non-remission in patients who exhibited poor early improvement (*COMT, PRNP*, *BRPF3*, *SLC25A40,* and *CGREF1* in males; *PPFIBPI*, *LZTS3, MEPCE, MAP1A,* and *PFAS* in females; *ST3GAL5* in the total population) were determined. Among the significant genes, variants in the *PRNP* (rs1800014), *COMT* (rs6267), *BRPF3* (rs200565609), and *SLC25A40* genes (rs3213633) were identified. However, interpretations should be made cautiously, as complex pharmacotherapy involves various genes and pathways. Early detection of poor early improvement and final non-remission based on genetic risk would be helpful for decision-making in a clinical setting.

## 1. Introduction

Depression is a prevalent mental disorder with a high disease burden. The mainstay of depression treatment is antidepressants, but high interindividual variability in response to antidepressants is a major obstacle for clinicians in selecting antidepressants in a clinical setting. Only one-third of patients entered remission after the first prescription of antidepressants. An additional one-third of patients exhibited improvements after a change to combination or augmentation therapy, and the remaining one-third failed to respond after being treated with at least two antidepressants with different mechanisms [1,2]. Effective antidepressants can be identified based only on trial and error. However, the recommended 4–8-week period for changing antidepressants delays recovery and contributes to a higher disease burden [3].

There has been considerable research effort put into identifying genetic markers for the response to antidepressants because genetic factors substantially contribute (42–50%) to interindividual variability in antidepressant response [4,5]. Previous candidate gene studies identified plausible polymorphisms underlying antidepressant mechanisms [6,7]. However, these studies had limitations in that limited variants were evaluated based on a partial understanding of antidepressant responses due to their hypothesis-driven design, and the findings were controversial. Moreover, they did not reflect the complex nature of antidepressant responses, in which many different genes are involved with multiple interactions. However, even in genome-wide association studies (GWAS) to identify novel genetic variants in hypothesis-free approaches, findings were inconclusive with no genome-wide significance and a lack of replication [1,8,9,10,11] even after combined analysis [12,13]. This may be due to sampling heterogeneity (study design and phenotype definition), the necessity for a large sample size for sufficient power, and limited methods targeting common and low-impact genetic variations. Whole-exome sequencing (WES), which has the advantage of being able to capture both rare and common variants in protein-coding regions, has the potential to address these unanswered questions. Previous sequencing studies indicated that the bone morphogenic protein gene was associated with poorer response to escitalopram [14], and one intergenic single nucleotide polymorphism (SNP) in chromosome 6 was associated with remission [15], but these studies had limited generalizability due to their small sample sizes.

Recent studies indicated that early improvement within 2 weeks of antidepressant treatment is a strong predictor of subsequent remission at 6–12 weeks [16,17], which supports the benefit of early clinical decision-making regarding subsequent treatment strategies. However, at least 20–30% of patients who did not exhibit early improvement also achieved remission after 4–12 weeks of treatment [18,19], which suggests that changing treatment options based on the early improvement status is premature. Therefore, identifying patients who exhibit poor early improvement within 2 weeks and eventual non-remission would be helpful for clinicians in terms of early clinical decision-making. However, there have been no investigations to identify genetic markers for subsequent non-remission in patients with poor early improvement status although genetic markers for early improvement and remission have been examined individually [8,12].

This study was performed to uncover genetic markers for predicting non-remission in patients exhibiting poor early improvement using WES data from a depression cohort (*n* = 1000) undergoing 12 weeks of antidepressant treatment with a naturalistic design. To decrease the limitation of sample heterogeneity, separate analyses were performed according to sex due to evidence indicating that there are differences between the sexes in terms of the effects of genetic vulnerabilities on depression risk [20] and antidepressant responses [21]. To consider the complex effects of multiple variants and their interactions on antidepressant response within genes as functional units, the gene-wise variant burden (GVB) scoring approach reflecting the cumulative contributions of several variants in specific genes [22] was used in the present analyses instead of polygenic risk scores, which do not yield reliable predictions of antidepressant response [23].

## 2. Results

### 2.1. Baseline Characteristics

Among 1262 depressive patients who participated in the MAKE BETTER study, 1000 patients consented to genetic testing and these comprised the final sample analyzed for the short-term treatment (12 weeks) outcome. Patients who did not undergo genetic testing were more likely to be unemployed (*p* = 0.003) and had a greater number of previous depressive episodes (*p* < 0.001), less severe suicidal ideation (*p* = 0.039), and more melancholic features (*p* = 0.037).

The recruitment and treatment processes are summarized in Supplementary Figure 1. Of the 1000 depressive patients treated naturalistically with antidepressants, 833 (83.3%) patients exhibited early improvement at 2 weeks post-baseline and 426 (42.6%) achieved remission at 12 weeks after baseline. Of the depressive patients who exhibited early improvement, 385 (46.2%) finally achieved remission at 12 weeks post-baseline, whereas 126 (75.4%) depressive patients who did not exhibit early improvement still had not achieved remission.

Supplementary Table S1 shows a comparison of baseline demographic and clinical characteristics between patients who exhibited poor early improvement and did not achieve final remission (*n* = 126) and those who exhibited early improvement and achieved final remission (*n* = 385).

### 2.2. Genes and Variants for Predicting Final Non-Remission after 12 Weeks of Treatment in Patients Exhibiting Poor Early Improvement at 2 Weeks

Two-step analyses at both the gene and variant levels were conducted. Well-known genes related to antidepressant mechanisms and novel genes were identified to predict final non-remission in patients who exhibited poor early improvement (Table 1). At the gene level (Table 2), more deleterious changes in Catechol-O-methyltransferase (*COMT*), Prion protein (*PRNP*), Bromodomain, PHD finger containing 3 (*BRPF3*), and Solute carrier family 25 member 40 (*SLC25A40*) genes according to multiple logistic regression analyses and cell growth regulator with EF-hand domain 1 (*CGREF1*) genes according to the SKAT-O test were associated with final non-remission only among male depressive patients who exhibited poor early improvement. Defective changes in PPFIA binding protein 1 (*PPFIBP 1*), leucine zipper tumor suppressor family member 3 (*LZTS3*), microtubule-associated protein 1A (*MAP1A*), methylphosphate capping enzyme (*MEPCE*), and phosphoribosylformylglycinamidine synthase (*PFAS*) genes also predicted final non-remission in female patients who exhibited poor early improvement according to the SKAT-O test. In addition, deleterious changes in the ST3 beta-galactoside alpha-2,3-sialyltransferase 5 (*ST3GAL5*) gene predicted final non-remission in all patients who exhibited poor early improvement, although no associations were seen in men or women separately. All associations were statistically significant even after correcting for multiple testing (FDR < 0.25).

At the variant level (Table 3), male depressive patients with altered known variants of rs1800014 in the *PRNP* gene and rs6267 in the *COMT* gene and with novel variants of rs200565609 in the *BRPF3* gene and rs3213633 in the *SLC25A40* gene were more likely to exhibit poor early improvement at 2 weeks and non-remission at 12 weeks. No specific variants were found to predict final non-remission with a poor early improvement in female patients.

### 2.3. Pathway Analyses

**Figure 1:**
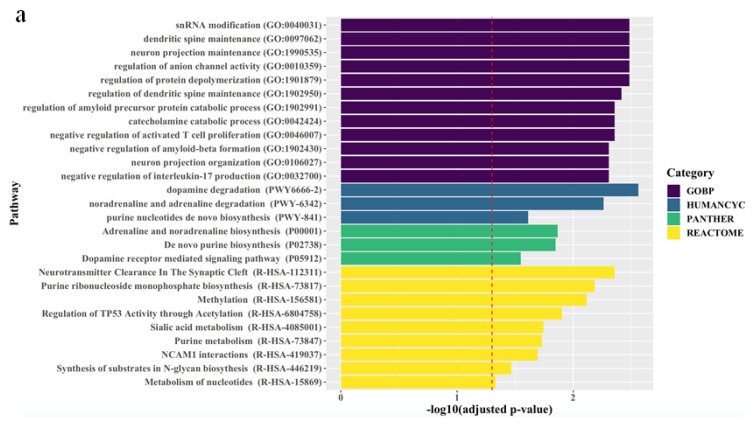

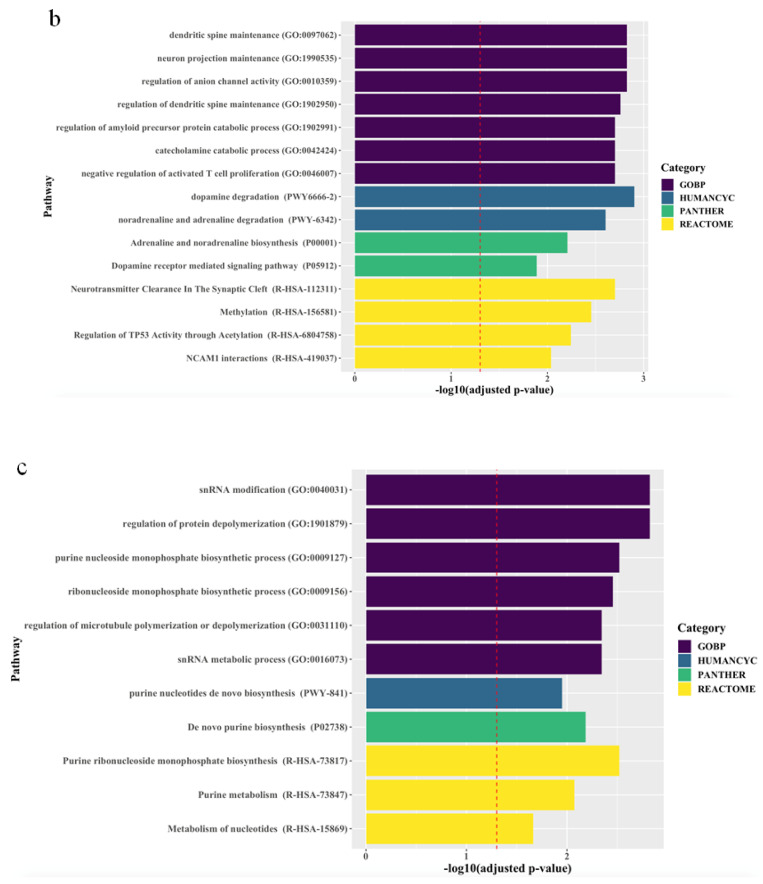
Top ranking candidate genes according to Gene Ontology with Biological Pathway (GO-BP), HumanCyc, Panther, and Reactome analyses. After adjustment using the Benjamini–Hochberg method, an adjusted *p* < 0.05 was taken to denote statistical significance. The results are ordered by significance in all categories. The red dashed vertical line represents adjusted *p* = 0.05, and (**a**), (**b**), and (**c**) indicate results in the total population, men, and women, respectively.

The associations between the biological pathways derived from the genes according to biological databases and all treatment outcomes are described in Figure 1. Pathway analyses revealed that, for all patients, final non-remission in patients exhibiting poor improvement at 2 weeks was associated with 27 pathways related to neuronal maintenance, neurotransmitter clearance and metabolism, and inflammatory and epigenetic mechanisms, even after correcting for multiple testing. Although there were no marked differences between men and women, genes predicting final non-remission in male patients exhibiting poor early improvement were likely to be enriched in direct modulation pathways related to neuronal maintenance and neurotransmitter metabolism, whereas genes predicting final non-remission in female patients exhibiting poor early improvement tended to be involved in indirect modulation pathways, including that for purine metabolism that mediates the neural release of monoamine and glutamate [24,25].

## 3. Methods

### 3.1. Study Outline

Data were obtained from the MAKE Biomarker Discovery for Enhancing anTidepressant Treatment Effect and Response (MAKE BETTER) Study, a 2-year prospective naturalistic study investigating markers that predict responses to antidepressants in real-world settings. The detailed study outline was published previously [26] and registered in cris.nih.go.kr (identifier: KCT0001332). Briefly, patients with depressive disorders were recruited, and antidepressants were prescribed according to the clinician’s judgment. Flexible dosages of antidepressants and augmenting agents were permitted. Co-medications, including other psychotropics (benzodiazepines and zolpidem) and other medications for concurrent medical conditions (antihypertensive and anti-diabetes drugs), were also allowed to reflect a real-world clinical situation. The baseline data collected at enrollment as well as follow-up data over 12 weeks for treatment outcomes were included in the present analyses. Written informed consent was obtained, and the study was approved by the Chonnam National University Hospital (CNUH) Institutional Review Board (Gwangju, South Korea; IRB code CNUH 2012-014 approved at 6 March 2012).

### 3.2. Participants, Antidepressant Treatment, and Outcomes

Participants were consecutively recruited from patients with depression who visited the psychiatric department of CNUH. Patients diagnosed by the study psychiatrists as having major depressive disorders, dysthymic disorders, and depressive disorders not otherwise specified using the Mini-International Neuropsychiatric Interview based on the DSM-IV criteria [27], and also with a score ≥ 14 on the Hamilton Depression Rating Scale (HAM-D) [28] were eligible for the MAKE BETTER study. Detailed inclusion and exclusion criteria are provided in the Supplementary Methods. Of the 1262 patients who were eligible and consented to participate in the study, 1000 agreed to genetic testing and thus comprised the sample for these analyses. All participants were Korean, who were ethnically homogenous.

Patients were treated by the study psychiatrists based on guidelines for the management of depressive disorders [29,30]. Specifically, monotherapy with first-line antidepressants was applied according to the clinician’s judgment for 3 weeks. Then, patients chose to either maintain the monotherapy, switch to another antidepressant, or combine antidepressants, with prescription of adjunctive medications, including antipsychotics and lithium, for 3, 6, or 9 weeks according to each patient’s preference while considering the naturalistic study design. The decision regarding strategies by patients was made after they had received guidance on the related factors by the study psychiatrists. Treatment outcomes were evaluated by measuring depression severity using the HAM-D at 1-, 2-, 3-, 6-, 9-, and 12-week follow-up assessments. Adherence was estimated based on pill counts at each visit, and depressive patients with acceptable adherence (at least 75%) were included in the present analyses.

To identify genetic risk factors for final non-remission in patients exhibiting poor early improvement, early improvement was defined as a ≥20% reduction in HAM-D score after 2 weeks of antidepressant treatment, whereas remission was defined as a HAM-D score ≤ 7 at 12 weeks, consistent with previous depression studies [31].

### 3.3. Demographic and Clinical Characteristics

Demographic and clinical characteristics potentially associated with the response to antidepressants were considered in the present analyses. Demographic data included age, marital status, years of education, employment status, and the number of chronic physical disorders. Clinical characteristics of the depressive disorders included the number of depressive episodes, onset age, duration of current episode, family history of depressive disorders, history of suicide attempts, baseline depression severity according to the HAM-D (Hamilton, 1960), the severity of anxiety symptoms according to the anxiety subscale of the Hospital Anxiety Depression Scale (HADS-A) [32], the severity of suicidal ideation according to the suicide-related items on the Brief Psychiatric Rating Scale (BPRS) [33], and specific depression subtypes, including melancholic, atypical, and psychotic features based on the DSM-IV criteria [34]. The HAMD, HADS, and BPRS scales have all been translated into Korean and validated [35,36,37]. Data on the treatment characteristics were further evaluated according to drug class, which included selective serotonin reuptake inhibitors (escitalopram, paroxetine, sertraline, and fluoxetine), serotonergic norepinephrine reuptake inhibitors (venlafaxine, duloxetine, and desvenlafaxine), and noradrenergic and specific serotonergic antidepressants (mirtazapine) and other drugs (bupropion, vortioxetine, and tricyclic antidepressants), as well as treatment strategies, which included antidepressant monotherapy, changes in or combined antidepressant therapy, augmentation, and combinations of the above therapies.

### 3.4. WES

DNA was extracted from venous blood samples from the 1000 MAKE BETTER participants who consented to genetic testing. Exomes were captured using an Agilent SureSelect Human All Exon V5-UTR kit (Agilent Technologies, Santa Clara, CA, USA), and then sequencing was performed (HiSeq2500; Illumina, San Diego, CA, USA) in the paired-end mode for 100- or 150-bp reads according to the manufacturer’s standard protocols. Detailed procedures are described in the Supplementary Methods. The mean coverage depth was 92.96 × and the mapping rate was 99.6%.

Captured exomes were processed using the bioinformatics pipeline following the best practice recommendations in GATK 3.3-0 [38]. Reads were mapped to the human genome reference sequence (hg19/GRCh37) using BWA 0.7.5a [39]. Duplicate reads were flagged using Picard Tools 1.101 (http://picard.sourceforge.net). GATK was used for short insertion and deletion (InDels) realignment, base quality score recalibration (BQSR), and finally, single-nucleotide variant and InDel discovery using Haplotype Caller across all samples simultaneously [38,40,41]. Variants were annotated with SnpEff 4.2 [42] and SnpSift 4.2 [43] software using dbNSFP 2.9.1 and Ensembl GRCh37.75.

### 3.5. Statistical Analysis

Depressive patients assessed at least both within the first 2 weeks and 3 weeks after baseline were included in the analyses due to the unavailability of defining treatment outcomes, including early improvement and remission. Multiple imputations by chained equations were used to estimate missing HAM-D scores after the second visit (at 3 weeks), according to age, sex, baseline HAM-D score, and baseline HADS-A score. To identify genes and variants with the potential for predicting final non-remission at 12 weeks in patients who exhibited poor early improvement at 2 weeks, it may be informative to compare genetic architecture at the extreme ends of the patient-outcome range (i.e., patients who exhibited early improvement at 2 weeks of antidepressant treatment and finally achieved remission at 12 weeks vs. patients who did not exhibit any early improvement and did not achieve eventual remission). Accordingly, the baseline demographic and clinical characteristics of patients who exhibited non-remission and poor early improvement were compared to those who achieved final remission and exhibited early improvement using the *t*-test or χ^2^ test.

**Figure 2:**
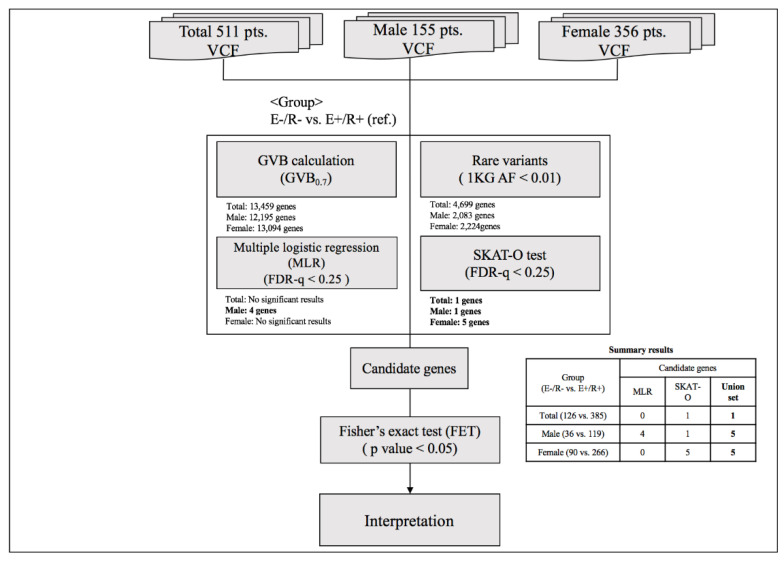
Summary of the analytic process. Abbreviations: VCF, Variant Call Format; GVB, gene-wise variant burden; 1 KG AF, Allele frequency of 1000 Genome; E(−)/R(−), later non-remission group in patients exhibiting poor early improvement; E(+)/R(+), later remission group in patients exhibiting early improvement, FDR, False discovery rate.

To identify genetic markers for final non-remission in patients who exhibited poor early improvement, two-step analyses were conducted as described in Figure 2. In the first-step analysis, a GVB scoring approach was used to estimate the cumulative impact of all deleterious variants, such as common, rare, and even novel genetic variants belonging to overlapping genes. The GVB approach aggregates the impacts of deleterious variants by combining the probabilities of the estimated likelihood of altered protein function. The GVB score is defined as the geometric mean of the Sorting Intolerant from Tolerant (SIFT) scores for all the deleterious variants in a gene [22]. Thus, the score ranges from 0 to 1, with lower scores representing a more deleterious impact as with the SIFT score [44]. This promising analytical approach was applied to identify genetic markers associated with the adverse effects of various drugs using WES [45,46]. The GVB values were compared between patients who exhibited early improvement and eventually achieved remission and those who did not exhibit early improvement and did not achieve remission at 12 weeks using multiple logistic regression analysis after adjustment for potentially significant demographic and clinical characteristics that may affect treatment response [1,47]. To compensate for the effects of rare variants, additional analyses were performed using the SNP-set/Sequence Kernel Association Test-optimal (SKAT-O) test [48]. Within individual genes, sets of rare variants, defined as allele frequency < 0.01 in the 1000 Genomes Project phase 3 data [49], were identified and the proportions of variant carriers were compared according to three treatment outcomes using the SKAT-O test. False discovery rate (FDR)-adjusted *p*-values were obtained with the Benjamini–Hochberg method to compensate for multiple statistical analyses. In the second-step analyses to identify genetic variants that predict final non-remission in patients exhibiting poor early improvement, individual variants within candidate genes exhibiting statistical significance (FDR < 0.25 either in the multiple logistic regression model or SKAT-O model) were compared between patients exhibiting early improvement who eventually achieved remission and those who did not exhibit early improvement nor achieve remission using Fisher’s exact test.

All statistical analyses were conducted using R 3.5.3 (http://www.r-project.org/). As genetic vulnerabilities in depression risk and antidepressant response have been shown to be different between sexes [20,21], all analyses were conducted on the total population and separately on men and women.

### 3.6. Pathway Analyses

To discover enriched function-related gene groups, pathway analysis was carried out with the pathway analysis tool Enrichr in R (http://amp.pharm.mssm.edu/Enrichr/) [50], which is a web-based tool to integrate analysis tools and biological databases, including Gene Ontology (http://www.geneontology.org/) enrichment analysis [51], Panther (http://www.pantherdb.org/pathway/) [52], Reactome (http://www.reactome.org/download) [53], and HumanCyc (https://humancyc.org/) [54], and provide a comprehensive list of functional annotations of genes to extract biological information. All genes implicated for non-remission in patients with poor early improvement status in the present study (FDR < 0.25) were considered for pathway analyses with Gene Ontology Biological Process, Panther, Reactome, and HumanCyc. All analyses were performed on the total population, and also separately on men and women. The Benjamini–Hochberg method was used to adjust the FDR in multiple testing. In all analyses, an adjusted *p* < 0.05 was taken to indicate statistical significance.

## 4. Discussion

Genetic markers predicting final non-remission after 12 weeks of antidepressant treatment in patients exhibiting poor early improvement at 2 weeks were found using the WES data from a naturalistically treated depression cohort—the well-known *COMT* gene and 10 novel genes associated with neural functions, including neuronal maintenance and neural transmission. Although contributing variants of four significant genes were identified, the cumulative impact of all deleterious variants at the gene level was more likely to contribute to final non-remission in patients exhibiting poor early improvement than the impact of any individual variant. In addition, different genes were associated with predicting final non-remission in male and female patients exhibiting poor early improvement at 2 weeks after 12 weeks of antidepressant treatment.

To our knowledge, this is the first modest-sized WES study to evaluate genetic markers associated with antidepressant response. We comprehensively investigated cumulative gene-level associations and then performed variant-level analyses on genes exhibiting statistical significance because variants do not act as single units but interact with one another within the same gene. Using this method, 11 genes were identified and shown to be enriched in the pathways related to neural plasticity, neurotransmitter metabolism, inflammatory response, and epigenetic modification, which are associated with depression treatment [55,56]. Notably, a few contributing variants were finally elucidated in many clinically meaningful genes in our cumulative gene-level analyses. These findings supported the concept that single variants alone may not have appreciable effects, but that combinations with other variants at the gene level may have the potential to explain treatment outcomes; thus, these variants represent complex traits. Although aggregating approaches at the gene, pathway, and polygenic risk score levels were recommended for future pharmacogenetic studies [23], further WES investigations with large samples are needed.

Genetic markers capable of predicting final non-remission after 12 weeks of antidepressant treatment in patients exhibiting poor early improvement at 2 weeks were found in the present study. There have been no previous studies regarding genetic markers for predicting remission based on early improvement status, although individual efforts have been made to identify genetic markers for early improvement and remission alone. Previous GWAS found potential markers (rs6989467 in the *CDH17* gene, *p* = 7.6 × 10^−7^; rs12054895 in the intergenic region of chromosome 5, *p* = 2.65 × 10^−8^) of early improvement [8,12], but these markers could not explain later remission. Based on the predictive role of early improvement in later remission [16,17], better treatment outcomes were observed when treatment strategies were changed according to early improvement status [57]. Considering the remission rate of 20–30% in patients exhibiting poor early improvement, our genetic markers, which predict final non-remission in patients exhibiting poor early improvement, may contribute to more accurate early decision-making regarding treatment options, which in turn would result in more effective medical treatment. Further prospective trials of cost-effectiveness of early treatment decisions based on genetic risk factors are needed.

Notably, different genes were shown to be associated with predicting final non-remission in male and female patients exhibiting poor early improvement. Among patients exhibiting poor early improvement, the risk of final non-remission was higher in male patients with *COMT*, *PRNP*, *BRPF3*, *SLC25A40,* and *CGREF1* genes with impaired function or harboring deleterious variants and in female patients with *PPFIBP1, LZTS3, MEPCE, MEP1A,* and *PFAS* genes with impaired function. Depressive patients exhibiting poor early improvement at 2 weeks with deleterious changes in the *ST3GAL5* gene were likely to not achieve eventual remission regardless of sex. Interestingly, risk-associated genes identified in males were common variants, whereas those in females and the total population were rare variants. These findings suggest that male depressive patients may be a more homogenous group than are female depressive patients, as reported previously in treatment studies [58]. Therefore, low-impact and common variants may contribute to the association between early improvement status and final remission status in male patients. By contrast, female depressive patients were heterogeneous with respect to treatment response because various biological and environmental factors are known to affect antidepressant response [58]. Therefore, high-impact and rare variants may impact treatment outcomes more significantly in women. There have been few previous GWAS and WES studies on sex-stratified associations with the antidepressant response, and future sex-stratified studies with larger cohorts composed of various ethnic populations are needed.

The implications of individually identified genes are additionally described in Supplementary Table S2. Of the identified genes and associated variants, *COMT* is the only gene previously evaluated as a predictor of the antidepressant response in candidate gene studies, based on its role in monoamine breakdown [59] and its interaction with serotonin and dopamine [60]. Among the *COMT* gene variants, the *Val158Met* (rs4680) polymorphism has been widely investigated. Significant associations with the antidepressant response and sex-specific associations have been reported, but the findings are inconsistent between studies [7,61] (see Supplementary Table S2). However, previous GWAS did not elucidate any significant associations between the antidepressant response and genes related to well-known mechanisms of antidepressants, including *COMT*. In our WES study, impaired COMT function or the deleterious *Ala72Ser* (rs6267) variant in the *COMT* gene was associated with later non-remission in patients exhibiting poor early improvement. The altered T allele (*Ser^7^*^2^) is known to be associated with reduced COMT activity [62], but no previous studies have investigated its relationship with the antidepressant response. Similar to our findings regarding COMT, the *Ala72Ser* (rs6267) variant has been consistently reported to be associated with a sex-specific increased risk of schizophrenia and the effects of treatment on negative symptoms [63,64]. Although the mechanisms underlying the sex-differential effects of an impaired *COMT* gene or the deleterious *Ala72Ser* (rs6267) variant have not been evaluated, the *COMT* gene has different impacts on brain function according to sex [65], and COMT activity is modulated by sex hormones [66]. Based on these findings, impaired COMT function due to changes in genes may affect monoamine concentrations via the α2-autoreceptor [67,68], contributing to a prolonged poor response to treatment and, thus, eventual non-remission in patients exhibiting poor early improvement. Meanwhile, this association might be representative of multiple genes and pathways associated with various psychotropics allowed in the present study with a naturalistic design. Other novel genes are suggested to be involved in maintaining the neural structure and neural transmission (see Supplementary Discussion and Supplementary Table S2) and may contribute to sustained non-remission after poor early improvement. Therefore, further investigations including measuring of expression levels and well-defined pharmacogenetic studies focusing on a single antidepressant are needed to understand the mechanisms underlying the roles of individual genes in predicting final non-remission in patients exhibiting poor early improvement and their sex-specific associations.

Several issues should be taken into consideration in the interpretation of these findings. First, the naturalistic study design can be both strength and a limitation. Naturalistic designs with broad inclusion and minimal exclusion criteria that allow treatment to be selected according to patient preference without a predetermined protocol, and the use of various drugs (antidepressants, other psychotropics including benzodiazepines, and co-medications for concurrent physical illness) and strategies, can maximize the generalizability of findings given the similarity to an actual clinical situation. A previous GWAS study used a naturalistic study design with heterogeneous antidepressant treatments to investigate genetic markers for treatment outcomes using a similar outcome definition for all antidepressants [8]. However, the minimal limitations in the study design may have affected the final treatment outcomes. Additionally, a lack of focus on a single type of treatment by allowing various psychotropics evidently obscured the findings due to the involvement of various genes and pathways. Therefore, care should be taken when interpreting the present findings. Further studies on the genetic predictors of specific antidepressants in well-defined studies with larger samples are required. Second, the small sample size of the present WES was not sufficient to identify variant frequency differences between patients exhibiting poor early improvement and final non-remission, and patients who exhibited early improvement and achieved final remission. To compensate for the limitation of sample size, we performed two-step analyses (the GVB test and variant analyses) and compared genetic architectures at the two extreme ends of the treatment outcomes (i.e., early improvement and final remission vs. poor early improvement and final non-remission). However, larger studies are needed to confirm our findings and provide additional statistical power. Third, recruitment was conducted at a single site and all participants were Korean. Further, no attempts were made to replicate the present findings within populations of the same or different ethnicities. Despite the consistency in evaluation and treatment, the single-center nature of the study and limitation to patients of East-Asian ethnicity may limit the generalizability of the present findings. However, this was the first study to investigate genetic factors for predicting treatment outcomes using WES data, and the results could serve as a foundation for future replication studies. Fourth, variants outside protein-coding regions were not included. Therefore, further research at the whole-genome level, including variants outside protein-coding regions, is required. Finally, in the present study, the early improvement was evaluated within 2 weeks of treatment. However, previous studies suggested early improvement even at 1 week of antidepressant treatment was predictive of subsequent remission [69,70]. Thus, future studies on early improvements at 1 week can help make decisions regarding antidepressant strategies during earlier periods.

In summary, genes with impaired function or deleterious gene variants that predict final remission status according to early improvement status were identified. Early treatment decisions based on the prediction of final remission status, which is in turn based on genetic architecture and early improvement status at 2 weeks, may help to reduce the duration of depressive symptoms and improve quality of life in depressive patients. Based on our findings, early improvement should be monitored closely in depressive patients with impaired candidate genes and variants. If patients exhibit poor early improvement, intensive treatment should be considered, including drug combinations and augmentation with other psychotropic agents early in the treatment period. Replication of our findings in larger multi-center settings using cohorts of various ethnicities may be needed to increase the generalizability of our findings as well as further evaluate differences among interventions according to genetic composition and casual interventions and their cost-effectiveness.

## Figures and Tables

**Table 1:** Summary of analysis results.

HUGO Gene Symbol	Description	Statistical Analysis	Response	Model		
			ER(−)/REM(−) Vs. ER(+)/REM(+)	Total	Male	Female
*PRNP*	Prion protein [HGNC:9449]	MLR	V		V	
*COMT*	Catechol-O-methyltransferase [HGNC:2228]		V		V	
*BRPF3*	Bromodomain and PHD finger containing 3 [HGNC:14256]		V		V	
*SLC25A40*	Solute carrier family 25 member 40 [HGNC:29680]		V		V	
*ST3GAL5*	ST3 Beta-Galactoside Alpha-2,3-Sialyltransferase 5 [HGNC:10872]		V	V		
*CGREF1*	Cell growth regulator with EF-hand domain 1 [HGNC:16962]	SKAT-O	V		V	
*PPFIBP1*	PPFIA binding protein 1 [HGNC:9249]		V			V
*LZTS3*	Leucine zipper tumor suppressor family member 3 [HGNC:30139]		V			V
*MAP1A*	Microtubule-associated protein 1A [HGNC:6835]		V			V
*MEPCE*	Methylphosphate capping enzyme [HGNC:20247]		V			V
*PFAS*	Phosphoribosylformylglycinamidine synthase [HGNC:8863]		V			V

MLR, Multiple Logistic Regression; ER(−)/REM(−), later non-remission group in patients exhibiting poor early improvement; ER(+)/REM(+), later remission group in patients exhibiting early improvement. V represents statistical significance with the correction of multiple hypothesis tests.

**Table 2:** Candidate genes for later non-remission in depressive patients exhibiting poor early improvement.

Analysis		MLR Result						SKAT-O	
	Group	Gene Symbol	*p*-Value	FDR q Value	OR (95% CI)	GVB (NR)	GVB (PR)	*p*-Value	FDR q Value
ER(−)/REM(−) vs. ER(+)/REM(+)	Total patients	*ST3GAL5*	NA	NA	NA	1.0 ± 0	1.0 ± 0	5.4 × 10^−5^	0.205 *
	Male patients	*BRPF3*	0.00056	0.2459 *	1.71 (3.41−8.63)	0.76 ± 0.4	0.91 ± 0.27	NA	NA
		*COMT*	0.00054	0.2459 *	8.72 (2.55−29.79)	0.59 ± 0.50	0.80 ± 0.40	NA	NA
		*SLC25A40*	0.00050	0.2459 *	14.84 (3.25−67.82)	0.70 ± 0.43	0.91 ± 0.27	NA	NA
		*PRNP*	0.00035	0.2459 *	9.43 (2.76−32.23)	0.62 ± 0.48	0.85 ± 0.35	NA	NA
		*CGREF1*	0.5250	1.0000	1.77 (0.12−4.73)	0.52 ± 0.47	0.47 ± 0.45	4.8 × 10^−5^	0.125 *
	Female patients	*PPFIBP1*	0.00782	0.3822	13.49 (1.98−91.79)	0.98 ± 0.18	0.98 ± 0.12	9.3 × 10^−6^	0.018 *
		*LZTS3*	NA	NA	NA	1.0 ± 0	1.0 ± 0	5.7 × 10^−5^	0.078 *
		*MEPCE*	NA	NA	NA	1.0 ± 0	1.0 ± 0	2.2 × 10^−4^	0.152 *
		*MAP1A*	0.60242	1.0000	1.45 (0.62−4.24)	0.50 ± 0.29	0.52 ± 0.29	2.3 × 10^−6^	0.009 *
		*PFAS*	0.87284	1.0000	0.99 (0.5−1.72)	0.37 ± 0.39	0.39 ± 0.37	1.9 ×10^−4^	0.150 *

MLR, Multiple Logistic Regression; NR, Negative Responder; PR, Positive Responder; GVB, Gene-wise Variant Burden (Mean); ER(−)/REM(−), later non-remission group in patients exhibiting poor early improvement; ER(+)/REM(+), later remission group in patients exhibiting early improvement; NA, Not applicable for the statistical test. (*) represents statistical significance with the correction of multiple hypothesis tests.

**Table 3:** Significant variants in candidate genes for later non-remission in depressive patients exhibiting poor early improvement.

Analysis		Additional FET Results															
Group		Gene Symbol	Position	Alt	rsID	SIFT	CADD	NR			PR			*p*-Value	OR (95% CI)	EAS AF	1 KG AF
								Ref	Het	Hom	Ref	Het	Hom				
Male patients	ER(−)/REM(−) vs. ER(+)/REM(+)	PRNP	chr20:4680521	G > A	rs1800014	0.03	14.46	26	9	1	108	11	0	0.00982	3.78 (1.45−9.84)	0.025	0.016
		COMT	chr22:19950263	G > T	rs6267	0.01	24.1	21	12	3	95	23	1	0.01492	2.83 (1.27−6.29)	0.035	0.013
		BRPF3	chr6:36168614	G > A	rs200565609	0.27	15.42	33	3	0	119	0	0	0.01173	Inf (NA-Inf)	0.001	0.00019
		SLC25A40	chr7:87476339	T > G	rs3213633	0.07	23.1	27	9	0	108	10	1	0.02157	3.27 (1.23−8.69)	0.052	0.011

FET, Fisher’s exact test; Alt, Alternative allele; rsID, Reference ID; SIFT, Sorting Intolerant From Tolerant; CADD, Combined annotation dependent depletion; NR, Negative Responder; PR, Positive Responder; Ref, Reference allele carrier; Het, Heterozygous alteration allele carrier; Hom, Homozygous alteration allele carrier; EAS AF, Allele frequency of East Asian in 1000 Genome; 1KG AF, Allele frequency of 1000 Genome; ER(−)/REM(−), later non-remission group in patients exhibiting poor early improvement; ER(+)/REM(+), later remission group in patients exhibiting early improvement.

## Supplementary Materials

The following are available online at https://www.mdpi.com/1422-0067/21/14/4884/s1.

## References

[1] Trivedi M.H., Rush A., Wisniewski S.R., Nierenberg A.A., Warden D., Ritz L., Norquist G., Howland R.H., Lebowitz B., McGrath P.J. (2006). Evaluation of Outcomes with Citalopram for Depression Using Measurement-Based Care in STAR*D: Implications for Clinical Practice. Am. J. Psychiatr..

[2] Souery D., Serretti A., Calati R., Oswald P., Massat I., Konstantinidis A., Linotte S., Bollen J., Demyttenaere K., Kasper S. (2011). Switching Antidepressant Class Does Not Improve Response or Remission in Treatment-Resistant Depression. J. Clin. Psychopharmacol..

[3] Steimer W., Müller B., Leucht S., Kissling W. (2001). Pharmacogenetics: A new diagnostic tool in the management of antidepressive drug therapy. Clin. Chim. Acta.

[4] Crisafulli C., Fabbri C., Porcelli S., Drago A., Spina E., De Ronchi D., Serretti A. (2011). Pharmacogenetics of Antidepressants. Front. Pharmacol..

[5] Tansey K., Guipponi M., Hu X., Domenici E., Lewis G., Malafosse A., Wendland J.R., Lewis C.M., McGuffin P., Uher R. (2013). Contribution of Common Genetic Variants to Antidepressant Response. Biol. Psychiatr..

[6] Porcelli S., Fabbri C., Serretti A. (2012). Meta-analysis of serotonin transporter gene promoter polymorphism (*5-HTTLPR*) association with antidepressant efficacy. Eur. Neuropsychopharmacol..

[7] Niitsu T., Fabbri C., Bentini F., Serretti A. (2013). Pharmacogenetics in major depression: A comprehensive meta-analysis. Prog. Neuro-Psychopharmacol. Biol. Psychiatr..

[8] Ising M., Lucae S., Binder E.B., Bettecken T., Uhr M., Ripke S., Kohli M.A., Hennings J.M., Horstmann S., Kloiber S. (2009). A genomewide association study points to multiple loci that predict antidepressant drug treatment outcome in depression. Arch. Gen. Psychiatr..

[9] Uher R., Perroud N., Ng M.Y., Hauser J., Henigsberg N., Maier W., Mors O., Placentino A., Rietschel M., Souery D. (2010). Genome-Wide Pharmacogenetics of Antidepressant Response in the GENDEP Project. Am. J. Psychiatr..

[10] Sasayama D., Hiraishi A., Tatsumi M., Kamijima K., Ikeda M., Umene-Nakano W., Yoshimura R., Nakamura J., Iwata N., Kunugi H. (2012). Possible association of *CUX1* gene polymorphisms with antidepressant response in major depressive disorder. Pharmacogenomics J..

[11] Myung W., Kim J., Lim S.-W., Shim S., Won H.-H., Kim S., Kim S., Lee M.-S., Chang H.S., Kim J.-W. (2015). A genome-wide association study of antidepressant response in Koreans. Transl. Psychiatr..

[12] GENDEP Investigators, MARS Investigators, STAR*D Investigators (2013). Common Genetic Variation and Antidepressant Efficacy in Major Depressive Disorder: A Meta-Analysis of Three Genome-Wide Pharmacogenetic Studies. Am. J. Psychiatr..

[13] Biernacka J.M., Sangkuhl K., Jenkins G., Whaley R.M., Barman P., Batzler A., Altman R.B., Arolt V., Brockmoller J., Chen C.H. (2015). The International SSRI Pharmacogenomics Consortium (ISPC): A genome-wide association study of antidepressant treatment response. Transl. Psychiatr..

[14] Tammiste A., Jiang T., Fischer K., Magi R., Krjutškov K., Pettai K., Esko T., Li Y., Tansey K., Carroll L.S. (2013). Whole-exome sequencing identifies a polymorphism in the *BMP5* gene associated with SSRI treatment response in major depression. J. Psychopharmacol..

[15] Wong M., Dong C., Flores D.L., Ehrhart-Bornstein M., Bornstein S., Arcos-Burgos M., Licinio J. (2014). Clinical outcomes and genome-wide association for a brain methylation site in an antidepressant pharmacogenetics study in Mexican Americans. Am. J. Psychiatr..

[16] Szegedi A., Jansen W.T., Van Willigenburg A.P.P., Van Der Meulen E., Stassen H.H., Thase M.E. (2009). Early improvement in the first 2 weeks as a predictor of treatment outcome in patients with major depressive disorder: A meta-analysis including 6562 patients. J. Clin. Psychiatr..

[17] Wagner S., Engel A., Engelmann J., Herzog D.P., Dreimüller N., Müller M.B., Tadić A., Lieb K. (2017). Early improvement as a resilience signal predicting later remission to antidepressant treatment in patients with Major Depressive Disorder: Systematic review and meta-analysis. J. Psychiatr. Res..

[18] Uher R., Mors O., Rietschel M., Rajewska-Rager A., Petrović A., Zobel A., Henigsberg N., Mendlewicz J., Aitchison K.J., Farmer A. (2011). Early and Delayed Onset of Response to Antidepressants in Individual Trajectories of Change During Treatment of Major Depression: A secondary analysis of data from the Genome-Based Therapeutic Drugs for Depression (GENDEP) study. J. Clin. Psychiatr..

[19] Gorwood P.A., Bayle F., Vaiva G., Courtet P., Corruble E., Llorca P.-M. (2013). Is it worth assessing progress as early as week 2 to adapt antidepressive treatment strategy? Results from a study on agomelatine and a global meta-analysis. Eur. Psychiatr..

[20] Kang H.-J., Park Y., Yoo K.-H., Kim K.-T., Kim E.-S., Kim J.-W., Kim S.-W., Shin I.-S., Yoon J.-S., Kim J.-M. (2020). Sex differences in the genetic architecture of depression. Sci. Rep..

[21] Pitychoutis P.M., Zisaki A., Dalla C., Papadopoulou-Daifoti Z. (2010). Pharamacogenetic Insight into depression and antidepressant response: Does sex matter?. Curr. Pharm. Des..

[22] Lee K.H., Baik S.Y., Lee S.Y., Park C.H., Park P.J., Kim J.H. (2016). Genome Sequence Variability Predicts Drug Precautions and Withdrawals from the Market. PLoS ONE.

[23] Corponi F., Fabbri C., Pae C.-U. (2019). Pharmacogenetics and Depression: A Critical Perspective. Psychiatr. Investig..

[24] Boison D. (2007). Adenosine as a modulator of brain activity. Drug News Perspect..

[25] Boison D. (2008). Adenosine as a neuromodulator in neurological diseases. Curr. Opin. Pharmacol..

[26] Kang H.-J., Kim J.-W., Kim S.-Y., Kim S.-W., Shin H.-Y., Shin M.-G., Kim J.-M. (2018). The MAKE Biomarker Discovery for Enhancing anTidepressant Treatment Effect and Response (MAKE BETTER) Study: Design and Methodology. Psychiatr. Investig..

[27] Sheehan D.V., Lecrubier Y., Sheehan K.H., Amorim P., Janavs J., Weiller E., Hergueta T., Baker R., Dunbar G.C. (1998). The Mini-International Neuropsychiatric Interview (M.I.N.I.): The development and validation of a structured diagnostic psychiatric interview for DSM-IV and ICD-10. J. Clin. Psychiatr..

[28] Hamilton M. (1960). A RATING SCALE FOR DEPRESSION. J. Neurol. Neurosurg. Psychiatr..

[29] Cowie M.R. (2015). National Institute for Health and Care Excellence. Eur. Hear. J..

[30] American Psychiatric Association Treating Major Depressive Disorder; Practice Guideline for the Treatment of Patients with Major Depressive Disorder. http://psychiatryonline.org/pb/assets/raw/sitewide/practive_guidellines/guidelines/mdd.pdf..

[31] deVries A.N., Roest A.M., Bos E.H., Burgerhof J.G.M., van Loo H.M., de Jonge P. (2019). Predicting antidepressant Response by monitoring early improvement of individual symptoms of depression:Indivdual patient data meta-analysis. Br. J. Psychiatr..

[32] Zigmond A.S., Snaith R.P. (1983). The Hospital Anxiety and Depression Scale. Acta Psychiatr. Scand..

[33] Overall J.E., Gorham D.R. (1962). The brief psychiatric rating scale. Psychol. Rep..

[34] American Psychiatric Association (2000). Diagnostic and Statistical Manual of Mental Disorders.

[35] Oh S.M., Min K.J., Park D.B. (1999). A study on the standardization of the hospital anxiety and depression scale for Koreans: A comparison of normal, depressed and anxious groups. J. Korean Neuropsychiatr. Assoc..

[36] Yi J.S., Bae S.O., Ahn Y.M., Park D.B., Noh K.S., Shin H.K., Woo H.W., Lee H.S., Han S.I., Kim Y.S. (2005). Validity and reliability of the Korean version of the Hamilton Depression Rating Scale (K-HDRS). J. Korean Neuropsychiatr. Assoc..

[37] Kim M.-K., Lee B.-K., Jeon Y.-W. (2003). Reliability of Korean Brief Psychiatric Rating Scale(BPRS)—Comparison of interrater reliability between the two rating methods and correlation of BPRS and SCL-90 self-report test. Korean. J. Clin. Psychol..

[38] McKenna A., Hanna M., Banks E., Sivachenko A., Cibulskis K., Kernytsky A., Garimella K., Altshuler D., Gabriel S., Daly M. (2010). The Genome Analysis Toolkit: A MapReduce framework for analyzing next-generation DNA sequencing data. Genome Res..

[39] Li H., Durbin R. (2010). Fast and accurate long-read alignment with Burrows-Wheeler transform. Bioinformatics.

[40] DePristo M.A., Banks E., Poplin R., Garimella K.V., Maguire J.R., Hartl C., Philippakis A.A., Del Angel G., Rivas M.A., Hanna M. (2011). A framework for variation discovery and genotyping using next-generation DNA sequencing data. Nat. Genet..

[41] Van Der Auwera G.A., Carneiro M.O., Hartl C., Poplin R., Del Angel G., Levy-Moonshine A., Jordan T., Shakir K., Roazen D., Thibault J. (2013). From FastQ Data to High-Confidence Variant Calls: The Genome Analysis Toolkit Best Practices Pipeline. Curr. Protoc. Bioinform..

[42] Cingolani P., Platts A.E., Wang L.L., Coon M., Nguyen T., Wang L., Land S.J., Lu X., Ruden D.M. (2012). A program for annotating and predicting the effects of single nucleotide polymorphisms, SnpEff: SNPs in the genome of Drosophila melanogaster strain w1118; iso-2; iso-3. Fly.

[43] Cingolani P., Patel V.M., Coon M., Nguyen T., Land S.J., Ruden D.M., Lu X. (2012). Using Drosophila melanogaster as a Model for Genotoxic Chemical Mutational Studies with a New Program, SnpSift. Front. Genet..

[44] Ng P.C., Henikoff S. (2003). SIFT: Predicting amino acid changes that affect protein function. Nucleic Acids Res..

[45] Seo H., Kwon E.J., You Y.-A., Park Y., Min B.J., Yoo K., Hwang H.-S., Kim J., Kim Y.J. (2018). Deleterious genetic variants in ciliopathy genes increase risk of ritodrine-induced cardiac and pulmonary side effects. BMC Med. Genom..

[46] Park Y., Kim H., Choi J.Y., Yun S., Min B.-J., Seo M.-E., Im H.J., Kang H.J., Kim J.H. (2019). Star Allele-Based Haplotyping versus Gene-Wise Variant Burden Scoring for Predicting 6-Mercaptopurine Intolerance in Pediatric Acute Lymphoblastic Leukemia Patients. Front. Pharmacol..

[47] Kim J.-M., Kim S.-W., Stewart R.J., Kim S.-Y., Yoon J.-S., Jung S.-W., Lee M.-S., Yim H.-W., Jun T.-Y. (2011). Predictors of 12-week remission in a nationwide cohort of people with depressive disorders: The CRESCEND study. Hum. Psychopharmacol. Clin. Exp..

[48] Lee S., Wu M.C., Lin X. (2012). Optimal tests for rare variant effects in sequencing association studies. Biostatics.

[49] (2015). The 1000 Genomes Project Consortium A global reference for human genetic variation. Nature.

[50] Kuleshov M.V., Jones M.R., Rouillard A., Fernandez N.F., Duan Q., Wang Z., Koplev S., Jenkins S.L., Jagodnik K.M., Lachmann A. (2016). Enrichr: A comprehensive gene set enrichment analysis web server 2016 update. Nucleic Acids Res..

[51] Harris M.A., Clark J., Ireland A., Lomax J., Ashburner M., Foulger R., Eilbeck K., Lewis S., Marshall B., Mungall C. (2004). The Gene Ontology (GO) database and informatics resource. Nucleic Acids Res..

[52] Mi H., Thomas P. (2009). PANTHER Pathway: An Ontology-Based Pathway Database Coupled with Data Analysis Tools. Methods Mol. Biol..

[53] Joshi-Tope G., Gillespie M., Vastrik I., D’Eustachio P., Schmidt E., de Bono B., Jassal B., Gopinath G.R., Wu G.R., Mattews L. (2005). Reactome: A knowledgebase of biological pathways. Nucleic Acids Res..

[54] Romero P., Wagg J., Green M.L., Kaiser D., Krummenacker M., Karp P.D. (2004). Computational prediction of human metabolic pathways from the complete human genome. Genome Biol..

[55] Menke A., Klengel T., Binder E.B. (2012). Epigenetics, depression and antidepressant treatment. Curr. Pharm. Des..

[56] Price J.B., Bronars C., Erhardt S., Cullen K.R., Schwieler L., Bert M., Walder K., McGee S.L., Frye M., Tye S.J. (2018). Boenergetics and synaptic plasticity as potential targets for individualizing treatment for depression. Neurosci. Biobehav. Rev..

[57] Tadić A., Wachtlin D., Berger M., Braus D.F., Van Calker D., Dahmen N., Dreimüller N., Engel A., Gorbulev S., Helmreich I. (2016). Randomized controlled study of early medication change for non-improvers to antidepressant therapy in major depression—The EMC trial. Eur. Neuropsychopharmacol..

[58] Sramek J.J., Murphy M.F., Cutler N.R. (2016). Sex differenxces in the psychopharmacological treatment of depression. Dialogues Clin. Neurosci..

[59] Fabbri C., Zohar J., Serretti A. (2018). Pharmacogenetic tests to guide drug treatment in depression: Comparison of the available testing kits and clinical trials. Prog. Neuro-Psychopharmacol. Biol. Psychiatr..

[60] Lenox R.H., Frazer A., Davis C.D., Coyle K.I., Nemeroff J.T. (2002). Mechanism of action of antidepressants and mood stabilizers. Neuropsychopharmacology: The Fifth Generation of Progress.

[61] Tsai S.-J., Gau Y.-T.A., Hong C.-J., Liou Y.-J., Yu Y.W.-Y., Chen T.-J. (2009). Sexually dimorphic effect of catechol-O-methyltransferase val158met polymorphism on clinical response to fluoxetine in major depressive patients. J. Affect. Disord..

[62] Nackley A.G., Shabalina S.A., Lambert J.E., Conrad M.S., Gibson D.G., Spiridonov A.N., Satterfield S.K., Diatchenko L. (2009). Low Enzymatic Activity Haplotypes of the Human Catechol-O-Methyltransferase Gene: Enrichment for Marker SNPs. PLoS ONE.

[63] Lee S.G., Joo Y., Kim B., Chung S., Kim H.L., Lee I., Choi B., Kim C., Song K. (2005). Association of Ala72Ser polymorphism with COMT enzyme activity and the risk of schizophrenia in Koreans. Hum. Genet..

[64] Chen C.-Y., Yeh Y.-W., Kuo S.-C., Ho P.-S., Liang C.-S., Yen C.-H., Lu R.-B., Huang S.-Y. (2016). Catechol-O-methyltransferase gene variants may associate with negative symptom response and plasma concentrations of prolactin in schizophrenia after amisulpride treatment. Psychoneuroendocrinology.

[65] Tunbridge E.M., Harrison P.J., Warden D.R., Johnston C., Refsum H., Smith A. (2008). Polymorphisms in the catechol-O-methyltransferase (*COMT*) gene influence plasma total homocysteine levels. Am. J. Med Genet. Part B: Neuropsychiatr. Genet..

[66] Jiang H., Xie T., Ramsden D.B., Ho S.-L. (2003). Human catechol-O-methyltransferase down-regulation by estradiol. Neuropharmacology.

[67] Linnér L., Arborelius L., Nomikos G.G., Bertilsson L., Svensson T.H. (1999). Locus coeruleus neuronal activity and noradrenaline availability in the frontal cortex of rats chronically treated with imipramine: Effect of α2-adrenoceptor blockade. Biol. Psychiatr..

[68] Szegedi A., Rujescu D., Tadic A., Muller M.J., Kohnen R., Stassen H.H., Dahmen N. (2005). The catechol-O-methyltransferase Val108/158Met polymorphism affects short-term treatment response to mirtazapine, but not to paroxetine in major depression. Pharmacogenomics J..

[69] Leuchter A.F., Cook I.A., Marangell L.B., Gilmer W.S., Burgoyne K.S., Howland R.H., Trivedi M.H., Zisook S., Jain R., McCracken J.T. (2009). Comparative effectiveness of biomarkers and clinical indicators for predicting outcomes of SSRI treatment in Major Depressive Disorder: Results of the BRITE-MD study. Psychiatr. Res..

[70] Browning M., Kingslake J., Dourish C., Goodwin G.M., Harmer C., Dawson G.R. (2019). Predicting treatment response to antidepressant medication using early changes in emotional processing. Eur. Neuropsychopharmacol..

