# Supplementary material for: Genetic Markers for Later Remission in Response to Early Improvement of Antidepressants
Source: Int J Mol Sci. 2020 Jul 10;21(14):4884. doi: 10.3390/ijms21144884 (PMC7402334; doi:10.3390/ijms21144884)
Supplement: Supplementary file 1 [file ijms-21-04884-s001.pdf]

Article

Genetic markers for later remission in response to early improvement of antidepressants

Hee-Ju Kang[1#,] Ki-Tae Kim[2#], Kyung-Hun Yoo[3], Yoomi Park[3], Ju-Wan Kim[1], Sung-Wan Kim, [1] Il-Seon Shin[1], Ju Han Kim[3*#], Jae-Min Kim[1*#]

Supplementary information

SUPPLEMENTARY METHODS……………………………………………………………..2

Eligibility Criteria……………………………………………………………………2

Whole exome sequencing……………………………………………………………2

SUPPLEMENTARY FIGURE S1……..……………………………………………..4

SUPPLEMENTARY TABLE S1……..…………………… …………..…………..5

SUPPLEMENTARY TABLE S2………………..………………...……………………..8

Supplementary Methods

Eligibility criteria

For the MAKE BETTER study, inclusion criteria were as follows: i) aged older than 7 years; ii) diagnosed with major depressive disorder, dysthymic disorder, or depressive disorder not otherwise specified (NOS), as ascertained using the Mini-International Neuropsychiatric Interview [1]; iii) Hamilton Depression Rating Scale [2] score ≥ 14; iv) able to complete questionnaires, understand the objective of the study, and sign the informed consent form. Exclusion criteria were as follows: i) unstable or uncontrolled medical condition; ii) unable to complete the psychiatric assessment or comply with the medication regimen, due to a severe physical illness; iii) current or lifetime DSM-IV diagnosis of bipolar disorder, schizophrenia, schizoaffective disorder, schizophreniform disorder, psychotic disorder NOS, or other psychotic disorder; iv) history of organic psychosis, epilepsy, or seizure disorder; v) history of anticonvulsant treatment; vi) hospitalization for any psychiatric diagnosis except depressive disorder (e.g., alcohol/drug dependence); vii) electroconvulsive therapy for the current depressive episode; viii) pregnant or breastfeeding.

Whole exome sequencing

WES was performed to screen coding sequence regions across the entire genome using the Illumina HiSeq 2500 sequencer (Illumina, Inc., San Diego, CA), with standard protocols, as described in the manufacturer's instructions. The SureSelect Human All Exon V5+UTR probe set, which includes 359,555 exons of 21,522 genes and has a total targeted region of 75 Mb, was used. To generate standard exome capture libraries, the Agilent SureSelect Target

Enrichment protocol for Illumina paired-end sequencing libraries (ver. B.3, June 2015) was used, with 3 μg of input gDNA. DNA quantity and quality were measured using PicoGreen reagent and a Nanodrop spectrophotometer. Genomic DNA aliquots (1 μg) were fragmented using adaptive focused acoustic technology (Covaris). Fragmented DNA was repaired, an 'A' residue was ligated to the 3′ end, and Agilent adapters were then ligated to the fragments. Once ligation had been assessed, adapter ligated products were amplified by PCR and the final purified products were quantified using qPCR, based on the qPCR Quantification Protocol Guide, and quality was assessed using the Caliper LabChip High Sensitivity DNA kit (PerkinElmer, Inc. Hopkinton, MA). For exome capture, 250 ng of DNA library was mixed with hybridization buffer, blocking mix, RNase block, and 5 μl of SureSelect all exon capture library, according to the standard Agilent SureSelect Target Enrichment protocol. Hybridization to capture baits was conducted at 65°C using a heated thermal cycler lid option at 105°C for 24 h on a PCR machine. The captured DNA was then amplified, and the final purified products were quantified by qPCR using the qPCR Quantification Protocol Guide and quality assessed using TapeStation DNA screen tape (Agilent).

Reference for Supplementary Method

1. Sheehan, D.V.; Lecrubier, Y.; Sheehan, K.H.; Amorim, P.; Janavs, J.; Weiller, E.; Hergueta, T.; Baker, R.; Dunbar, G.C. The mini-international neuropsychiatric interview (M.I.N.I): the development and validation of a structured diagnostic psychiatric interview for DSM-IV and ICD-10. *J. Clin. Psychiatry* **1998**, 59, S22-S33.
2. Hamilton, M. A rating scale for depression. *J. Neurol. Neurosurg. Psychiatry* **1960**, 23, 56-62.

Supplementary Figure 1. Process of Recruitment

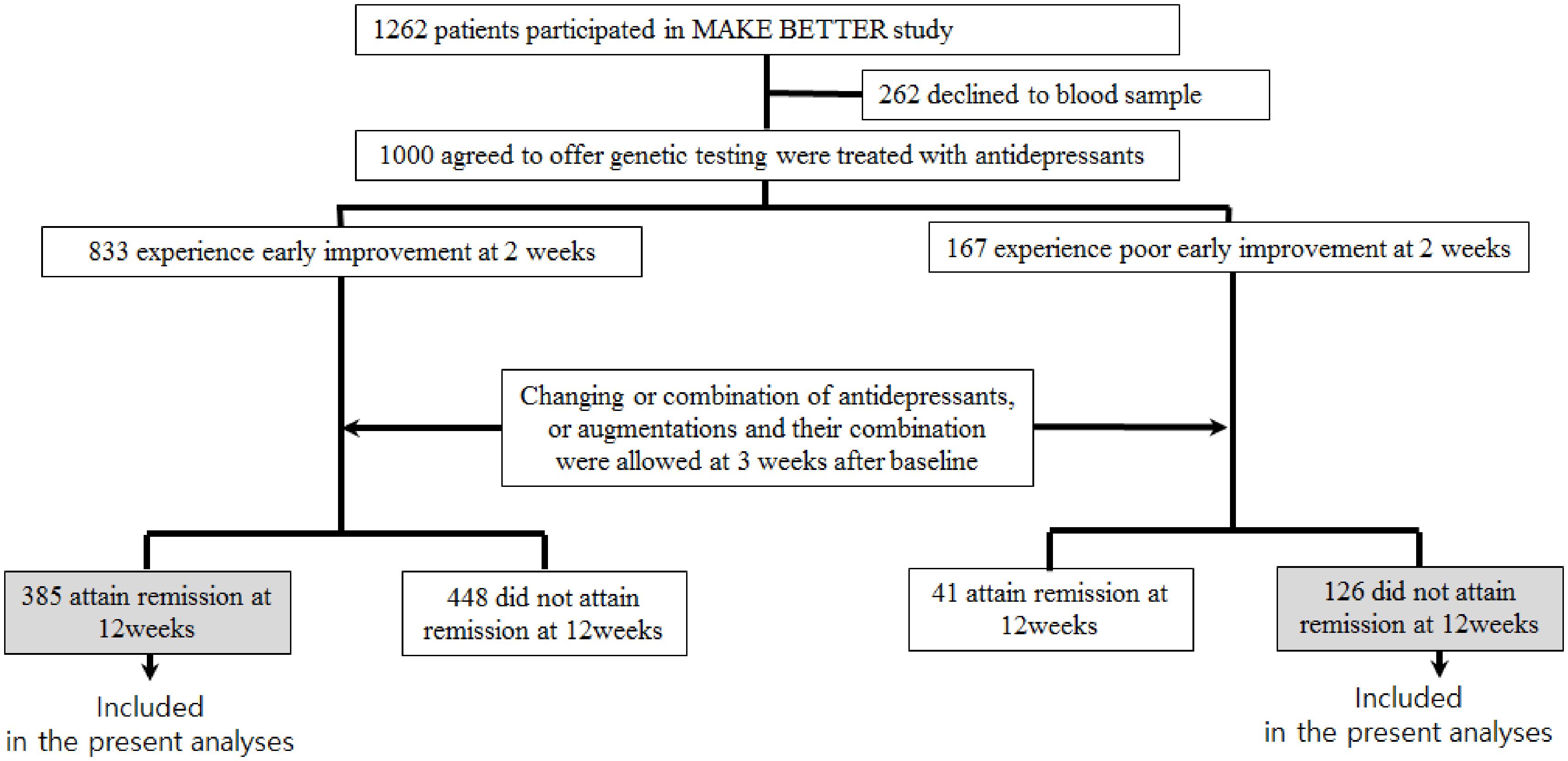

Supplementary Table S1. Demographic and clinical characteristics of patients who exhibited early improvement and later remission and those who exhibited poor early improvement with later non-remission

	Remission by early response status								
	Male (N=155)			Female (N=356)			Total (N=511)		
	ER(-)/ REM(-) (N=36)	ER(+) /REM(+) (N=119)	P-value	ER(-)/ REM(-) (N=90)	ER(+)/ REM(+) (N=266)	P-value	ER(-)/ REM(-) (N=126)	ER(+)/ REM(+) (N=385)	P-value
**Sociodemographic**									
Age,mean(SD), years	52.4 (18.8)	56.4 (14.6)	0.252	56.6 (14.7)	59.1 (13.1)	0.133	55.4 (16.0)	58.2 (13.6)	0.075
Sex, n(%), female	-	-	-	-	-	-	90 (71.4)	266 (69.1)	0.620
Education, mena(SD), years	11.1 (3.8)	11.3 (4.4)	0.818	8.1 (4.9)	7.9 (4.8)	0.705	9.0 (4.8)	9.0 (5.0)	0.960
Marital status, n(%), non-married	12 (33.3)	28 (23.5)	0.239	28 (31.1)	78 (29.3)	0.749	40 (31.7)	106 (27.5)	0.363
Unemployment status, n(%)	14 (38.9)	32 (26.9)	0.167	22 (24.4)	60 (22.6)	0.713	36 (28.6)	92 (23.9)	0.293
**Depression characteristics**									
Number of episode, mean(SD)	1.9 (5.9)	1.3 (3.4)	0.487	2.2 (4.2)	1.5 (3.4)	0.707	2.1 (4.7)	1.5 (3.4)	0.148
Age onset, mean(SD), years	49.9 (20.2)	53.1 (16.9)	0.350	49.9 (16.4)	54.1 (15.0)	**0.027**	49.9 (17.5)	53.8 (15.6)	**0.029**

Duration of illness, mean(SD), Day	336.9 (648.9)	219.4 (545.9)	0.224	536.8 (1332.1)	229.1 (398.4)	**0.033**	479.7 (1178.8)	226.1 (416.1)	**0.019**
Family history of depression, n(%), yes	6 (16.7)	16 (13.4)	0.627	13 (14.4)	45 (16.9)	0.583	19 (15.1)	61 (15.8)	0.838
History of suicide attempt, n(%), yes	6 (16.7)	15 (12.6)	0.533	10 (11.1)	17 (6.4)	0.144	16 (12.7)	32 (8.3)	0.143
HAMD baseline score, mean(SD)	18.2 (3.3)	19.9 (3.9)	**0.018**	19.3 (4.2)	20.6 (4.2)	**0.017**	19.0 (4.0)	20.4 (4.1)	**0.001**
HADS-anxiety subscale score, mean(SD)	12.9 (4.0)	11.2 (4.0)	**0.025**	12.5 (3.8)	11.1 (4.1)	**0.005**	12.6 (3.9)	11.1 (4.1)	**<0.001**
Suicide item of BPRS, n(%)									
Mild or less(1-3)	27 (75.0)	84 (70.6)	0.607	55 (61.1)	207 (77.8)	**0.002**	82 (65.)	291 (75.6)	**0.021**
Moderate to extreme severe (4-7)	9 (25.0)	35 (29.4)		35 (38.9)	59 (22.2)		44 (34.9)	94 (24.4)	
Features of depression, n(%)									
Atypical (yes)	2 (5.6)	7 (5.9)	1.000	8 (8.9)	16 (6.0)	0.347	10 (7.9)	23 (6.0)	0.437
Melancholic(yes)	2 (5.6)	24 (20.2)	**0.040**	11 (12.2)	3 (11.7)	0.885	13 (10.3)	55 (14.3)	0.255
Psychotic (yes)	5 (13.9)	3 (2.5)	**0.017**	7 (7.8)	13 (4.9)	0.303	12 (9.5)	16 (4.2)	**0.022**
Treatment									
Drug class			0.606			0.512			0.856
SSRI	26 (72.2)	74 (62.2)		49 (54.4)	167 (62.8)		75 (59.6)	241 (62.6)	

SNRI	3 (8.3)	9 (7.6)		11 (12.2)	28 (10.5)		14 (11.1)	37 (9.6)	
NaSSA	5 (13.9)	29 (24.4)		25 (27.8)	62 (23.3)		30 (23.8)	91 (23.6)	
Others	2 (5.6)	7 (5.9)		5 (5.6)	9 (3.4)		7 (5.6)	16 (4.2)	
Treatment strategy			0.382			0.684			0.286
monotherapy	18 (50.0)	40 (33.6)		37 (41.1)	101 (38.0)		55 (43.7)	141 (36.6)	
Switching	3 (8.3)	11 (9.2)		7 (7.8)	20 (7.5)		10 (7.9)	31 (8.1)	
Combination	5 (13.9)	14 (11.8)		16 (17.8)	36 (13.5)		21 (16.7)	50 (13.0)	
Augmentation	5 (13.9)	27 (22.7)		14 (15.6)	57 (21.4)		19 (15.1)	84 (21.8)	
2 or 3 of switching, combination, augmentation	5 (13.9)	27 (22.7)		16 (17.8)	52 (19.5)		21 (16.7)	79 (20.5)	
Number of physical illness, mean(SD)			0.876			0.740			
Absence	8 (22.2)	25 (21.0)		18 (20.0)	49 (18.4)		26 (20.6)	74 (19.2)	0.728
1 or more	28 (77.8)	94 (79.0)		72 (80.0)	217 (81.6)		100 (79.4)	311 (80.8)	
Menopause, n(%), yes	-	-	-	65 (72.2)	218 (82.0)	**0.048**	-	-	-

P-values were calculated using *t*-test, χ^2 test, or Fisher's exact tests, as appropriate.

Values in bold type show broader significance cut-off ($P < 0.05$).

ER(-)/REM(-), Both negative group in Early response and Remission; ER(+)/REM(+), Both positive group in Early response and Remission; HAMD, Hamilton Depression Rating Scale; HADS, Hospital Anxiety Depression Scale; BPRS, Brief Psychiatric Rating Scale; SSRI, selective Serotonin reuptake inhibitor; SNRI, Serotonergic norepinephrine reuptake inhibitor; NaSSA, noradrenergic and specific serotonergic antidepressant

Supplementary Table S2. Summary of previous findings regarding identified genes and variants

Gene	Variant	Function	Previous findings	Sex-specific findings
COMT	*Val158Met* (rs4680)	Encodes catechol-O-methyltransferase (COMT)<br>-degrades catecholamines, such as dopamine, epinephrine, and norepinephrine<br>-located in postsynaptic neurons<br>-Met allele associated with low COMT activity<br>-women exhibit low COMT activity [1]<br>-estradiol downregulates COMT expression and activity [2]	**Association with AD response widely investigated**<br>1) associated with better response [3-6]<br>2) associated with poor response [7,8]<br>3) no significant association [9,10]<br>➔ **controversial findings**	**AD response**<br>Male-specific [3]<br>Female-specific [4]<br><br>**Disease vulnerability**<br>Male-specific: obsessive-compulsive disorder (OCD), lethal suicide attempt, cognitive function [11-13]<br>Female-specific: OCD, panic, schizophrenia [14-16]<br>➔ **controversial findings**
	Ala72Ser (rs6267)	Encodes COMT<br>-*Ser* allele associated with low COMT activity	**Association with AD response not investigated**<br>Schizophrenia:<br>-*Ser* allele associated with increased schizophrenia risk<br>-*Ser* allele associated with better response of negative symptoms symptom [17,18]<br>Parkinson's disease:<br>-*Ser* allele associated with severe pain [19]	**Association with AD response not investigated**<br>Female specific-risk of schizophrenia [17]

PRNP	rs1800014	Encodes prion protein<br>Prion protein function<br>-neural protection: affects NMDA receptor in hippocampal neurons [20]<br>-synaptic functions: interacts with synaptic release proteins or neurotransmitter receptors [21,22]<br>-T cell activation [23]	**Association with AD response not investigated**<br>-depressive-like behavior in mice with impaired *PRNP* gene function [24]<br>-lower PRNP expression level in major depressive patients [25]<br><br>**rs1800014 alteration in *PRNP* gene**<br>-protective effect against sporadic Creutzfeldt–Jakob disease in Japan and Korea [26]<br>➔ lowered PRNP function related to neuronal protection and synaptic maintenance due to impaired *PRNP* gene function may prolong depressive symptoms and prevent antidepressant action	**Not investigated**
BRPF3	rs200565609	Encodes bromodomain-containing protein family IV<br>-epigenetic readers that recognize acetylation histone tails to facilitate transcription of target genes [27]<br>-highly expressed in brain<br>-expendable, loss does not affect survival [28]	**Association with AD response not investigated**<br>high sequence similarity to *BRPF2*<br>-*BRPF2*: linked to schizophrenia, bipolar disorder; deficiency results in lethality due to role in neurodevelopment [29]<br>➔ *BRPF3* genes may be involved in advanced brain functions, such as intellectual reasoning and affect regulation, which are not necessary for survival<br>Impaired affect regulation contributes to prolonged poor AD treatment outcomes.<br>➔Impaired epigenetic regulation by *BRPF3*	**Not investigated**

			affects AD response	
SCL25A40	rs3213633	Encodes mitochondrial carrier proteins that function as mitochondrial membrane transporters<br>-widely expressed in central nervous system mitochondria<br>-regulation of neurotransmission and neuronal degeneration	**Association with AD response not investigated**<br>-increased susceptibility to epilepsy and neurodegenerative disorders [30,31]<br>-SLC25A40 expression is reduced in chronically defeated mice [32]<br>-candidate gene for chronic fatigue [33]<br>➔ Impaired *SLC25A40* gene variants, including rs3213633, may result in mitochondrial dysfunction, leading to impaired neurotransmission that lowers antidepressant efficacy and final non-remission	**Not investigated**
CGREF1		Encodes a secretory protein involved in cell adhesion and proliferation<br>-widely expressed in the brain	**Association with AD response not investigated**<br>Risk-taking behavior in genotype specific-expression analyses [34]<br>➔ CGREF1 may be involved in synaptic plasticity, which confers the possibility of predicting final remission status[35]	**Not investigated**
LZTS3		Member of the gene family encoding leucine zipper tumor suppressor (LZTS)<br>-mainly expressed in the brain<br>-involved in regulation of postsynaptic interaction and maturation of dendritic	**Association with AD response not investigated**<br>Drives alcohol-drinking and -seeking behaviors [36]<br>➔ The role played by LZTS3 in synaptic regulation and increased alcohol dependence may make it a good predictor of AD response/remission status	**Not investigated**

	spines in the hippocampus		
MEPCE	-catalyzes the addition of a methyl phosphate cap to 7SK snRNA<br>-participates in transcriptional regulation at the transition from initiation to elongation<br>➔ Neuronal development and differentiation	**Association with AD response not investigated**<br>-neurodevelopmental disorders showing developmental delay, seizure, and visual impairment [37]<br>➔ Regulation of neural plasticity by *MEPCE* gene may influence AD treatment outcome	**Not investigated**
MEP1A	Encodes meprin α, a metalloprotease<br>-capable of cleaving a wide variety of substrates, including membrane proteins, protein kinases, and cytokines [38]<br>-lack of meprin α protein is associated with heightened inflammatory response	**Association with AD response not investigated**<br>-abnormal MEP1A expression has been implicated in several diseases, such as inflammatory bowel disease, nephritis, and Alzheimer's disease [38,39]<br>➔ Impaired *MEP1A* gene function leads to a prolonged inflammatory response, which results in poor early improvement and final non-remission despite AD treatment.	**Not investigated**<br>*MEP1A* has been suggested to be a target gene for abnormal glucose metabolism in polycystic ovary syndrome (PCOS), the most common endocrine disorder in women [40]<br>➔ Previous findings of MEP1A's role in PCOS patients also support our female-specific association with treatment outcomes.
PFAS	Encodes phosphoribosyl formylglycinamidine synthase, a core enzyme	**Association with AD response not investigated**<br>Purine biosynthesis:<br>-involved in maintaining intracellular energy	**Not investigated**

	involved in *de novo* purine synthesis	stores and neurotransmission [41]<br>-dysregulation of purinergic signaling has been linked to neurological diseases, including epilepsy, Alzheimer's disease [42] and depression [43]<br>-knockdown of *PFAS* is involved in sleep regulation and energy stores [44]<br>➔ Deleterious changes in the PFAS gene lead to sleep disturbance and lower energy levels that are included in depressive symptoms, and in turn, these symptoms may be sustained even with AD treatment.	
ST3GAL5	Encodes a sialyltransferase<br>-synthesizes ganglioside (GM3)<br>-modulates postsynaptic membrane functions in neural synapses	-a decrease in GM3 synthesis due to both homozygous and heterozygous genetic variants in *ST3GAL5* causes intellectual disabilities, choreoathetosis, and/or epilepsy [45,46]<br>-evidence suggests an association with bipolar disorders [47-49]<br>➔ Although an association with mood disorders (bipolar disorder and depression) without evident underlying mechanisms was suggested, genes related to membrane function may be involved in neuroplasticity, in turn resulting in vulnerabilities to mood disorders and treatment resistance.	**Not investigated**

AD, antidepressant

Reference for supplementary Table 2

1. Puzynski, S.; Bidzinski, A.; Mrozek, S.; Zaluska, M. Studies on biogenic amine metabolizing enzymes (DBH, COMT, MAO) andpathogenesis of affective illness. II. Erythrocyte catechol-O-methyltransferase activity in endogenous depression. *Acta Psychiatr. Scand*. **1983**, 67, 96–100.

2. Jiang, H.; Xie, T.; Ramsden, D.B.; Ho, S.L. Human catechol-O-methyltransferase down-regulation by estradiol. *Neuropharmacolog*y **2003**, 45, 1011–1018.

3. Tsai, S.J.; Gau, Y.T.A.; Hong, J.H.; Liou, Y.J.; Yu, Y.W.Y.; Chen, T.J. Sexually dimorphic effect of catechol-*O*-methyltransferase val158met polymorphism on clinical response to fluoxetine in major depressive patients. *J. Affect. Disord.* **2009**, 113, 183-187.

4. Baune, B.T.; Hohoff, C.; Berger, K.; Neumann, A.; Mortensen, S.; Roehrs, T.; Deckert, J.; Arolt, V.; Domschke, K. Association of the COMT val158met variant with antidepressant treatment response in major depression. *Neuropsychopharmacology* **2008**, 33, 924–932.

5. Benedetti, F.; Colombo, C.; Pirovano, A.; Marino, E.; Smeraldi, E. The catechol-O-methyltransferase Val(108/158)Met polymorphism affects antidepressant response to paroxetine in a naturalistic setting. *Psychopharmacology (Berl.)* **2009**, 203, 155–160.

6. Yoshida, K.; Higuchi, H.; Takahashi, H.; Kamata, M.; Sato, K.; Inoue, K.; Suzuki, T.; Itoh, K.; Ozaki, N. (2008). Influence of the tyrosine hydroxylase val81met polymorphism and catechol-O-methyltransferase val158met polymorphism on the antidepressant effect of milnacipran. *Hum. Psychopharmacol.* **2008,** 23, 121–128

7. Szegedi, A.; Rujescu, D.; Tadic, A.; Muller, M.J.; Kohnen, R.; Stassen, H.H.; Dahmen, N. The catechol-O-methyltransferase Val108/158Met polymorphism affects short-term treatment response to mirtazapine, but not to paroxetine in major depression. *Pharmacogenomics J.* **2005**, 5, 49–53.

8. Arias, B.; Serretti, A.; Lorenzi, C.; Gastó, C.; Catalán, R.; Fañanás, L. Analysis of COMT gene (Val 158 Met polymorphism) in the clinical response to SSRIs in depressive patients of European origin. *J. Affect. Disord.* **2006**, 90, 251–256.

9. Hilli, J.; Heikkinen, T.; Rontu, R.; Lehtimaki, T.; Kishida, I.; Aklillu, E.; Bertilsson, L.; Vahlberg, T.; Laine, K. MAO-A and COMT genotypes as possible regulators of perinatal serotonergic symptoms after in utero exposure to SSRIs. *Eur. Neuropsychopharmacol.* **2009,**. 19, 363–370.

10. Niitsu, T.; Fabbri, C.; Bentini, F.; Serretti, A. Pharmacogenetics in major depression: a comprehensive meta analysis. *Prog Neuropsychopharmacol Biol Psychiatry* **2013**, 45, 183-194.

11. Kia-Keating, B.M.; Glatt, S.J.; Tsuang, M.T. Meta-analyses suggest association between COMT, but not HTR1B, alleles, and suicidal behavior. *Am. J. Med. Genet. B Neuropsychiatr. Genet.* **2007,** 144B, 1048–1053.

12. Pooley, E.C.; Fineberg, N.; Harrison, P.J. The met(158) allele of catechol-O-methyltransferase (COMT) is associated with obsessive–compulsive disorder in men: case–control study and meta-analysis. *Mol. Psychiatry* **2007**, 12, 556–561.

13. Barnett, J.H.; Heron, J.; Ring, S.M.; Golding, J.; Goldman, D.; Xu, K.; Jones, P.B.

Gender-specific effects of the catechol-O-methyltransferase Val108/158Met polymorphism on cognitive function in children. *Am. J. Psychiatry* **2007,** 164, 142–149.

14. Alsobrook, II, J.P.; Zohar, A.H.; Leboyer, M.; Chabane, N.; Ebstein, R.P.; Pauls, D.L. Association between the COMT locus and obsessive–compulsive disorder in females but not males. *Am. J. Med. Genet.* **2002**, 114, 116–120.

15. Domschke, K.; Freitag, C.M.; Kuhlenbaumer, G.; Schirmacher, A.; Sand, P.; Nyhuis, P.; Jacob, C.; Fritze, J.; Franke, P.; Rietschel, M.; et al. Association of the functional V158M catechol-O-methyl-transferase polymorphism with panic disorder in women. *Int. J. Neuropsychopharmacol.* **2004**, 7, 183–188.

16. Kremer, I.; Pinto. M.; Murad, I.; Muhaheed, M.; Bannoura, I.; Muller, D.J.; Schulze, T.G.; Reshef, A.; Blanaru, M.; Gathas, S.; et al (2003). Family-based and case–control study of catechol-Omethyltransferase in schizophrenia among Palestinian Arabs. *Am. J. Med. Genet. Neuropsychiatr. Genet.* **2003**, 119, 35–39.

17. Lee, S.G.; Joo, Y.; Kim, B.; Chung, S.; Kim, H.L.; Lee, I.; Choi, B.; Kim, C.; Song, K. Association of Ala72Ser polymorphism with COMT enzyme activity and therisk of schizophrenia in Koreans. *Hum. Genet.* **2005**, 116, 319–328.

18. Chen, C.Y.; Yeh, Y.W.; Kuo, S.C.; Ho, P.S.; Liang, C.S.; Yen, C.H.; Lu, R.B.; Huang, S.Y. Catechol-*O*-methyltransferase gene variants may associated with negative symptom response and plasma concentration of prolactin in schizophrenia after amisulpride treatment. *Psychoendocrinology* **2016**, 65, 67-75.

19. Lin, C.-H.; Chaudhuri, K.R.; Fan, J.-Y.; Ko, C.-I.; Rizos, A.; Chang, C.-W.; Lin, H.-I.; Wu, Y.-R. Depression and catechol-O-methyltranferase (COMT) genetic variants are associated with pain in Parkinson's disease. *Sci. Rep.* **2017,** 7, 6306.

20. Khosravani, H.; Zhang, Y.; Tsutsui, S.; Hameed, S.; Altier, C.; Hamid, J.; Chen, L.; Villemaire, M.; Ali, Z.; Jirik, F.R.; et al. Prion protein attenuates excitotoxicity by inhibiting NMDA receptors. *J Cell Biol*. **2008,** 181, 551–565.

21. Roucou, X.; Gains, M.; LeBlanc, A.C. Neuroprotective functions of prion protein. *J Neurosci Res*. **2004**, 75, 153–161.

22. Mouillet-Richard, S.; Schneider, B.; Pradines, E.; Pietri, M.; Ermonval, M.; Grassi, J.; Richards, J.G.; Mutel, V.; Launay, J.M.; Kellermann, O. Cellular prion protein signaling in serotonergic neuronal cells. *Annals New York Acad. Sci.* **2007**, 1096, 106-119.

23. Ingram, R.J.; Isaacs, J.D.; Kaur, G.; Lowther, D.E.; Reynolds, C.J.; Boyton, R.J.; Collinge, J.; Jackson, G.S.; Altmann, D.M. A role of cellular prion protein in programming T-cell cytokine responses in disease. *FASEB J* **2009**, 23, 1672e84.

24. Gadotti, V.M.; Bonfield, S.P.; Zamponi, G.W. Depressive-like behaviour of mice lacking cellular prion protein. *Behav Brain Res*. **2012,** 227. 319–323.

25. Watanabe, S.Y.; Iga, J.I.; Ishii, K.; Numata, S.; Shimodera, S.; Fujita, H.; Ohmori, T. Biological tests for major depressive disorder that involve leukocyte gene expression assays. *J. Psychiatr. Res.* **2015**, 66-67, 1-6.

26. Shibuya, S.; Higuchi, J.; Shin, R.W.; Tateishi, J.; Kitamoto, T. Protective prion

protein polymorphisms against sporadic Creutzfeldt-Jakob disease. *Lancet.* **1998,** 351, 419.

27. Lloyd, J.T.; Class, K.C. Biologic function and histone recognition of family IV bromodomain-containing protiens. *J. Cell. Physiol.* **2018,** 233, 1877–1886
28. Yan, K.; You, L.; Degerny, C.; Ghorbani, M.; Liu, X.; Chen, L.; Li, L.; Miao, D.; Yang, X.J. The Chromatin Regulator BRPF3 Preferentially Activates the HBO1 Acetyltransferase but Is Dispensable for Mouse Development and Survival. *J. Biol. Chem.* **2016**, 291, 2647–2663.
29. Nyegaard, M.; Severinsen, J.E.; Als, T.D.; Hedemand, A.; Straarup, S.; Nordentoft, M.; McQuillin, A.; Bass, N.; Lawrence, J.; et al. Support of Association Between BRD1 and Both Schizophrenia and Bipolar Affective Disorder. *Am. J. Med. Genet. B.* **2010**, 153B(2), 582–591
30. Morais, V.A.; Verstreken. P.; Roethig, A.; Smet, J.; Snellinx, A.; Vanbrabant, M.; Haddad, D.; Frezza, C.; Mandemakers, W.; Vogt-Weisenhorn, D.; et al. Parkinson's disease mutations in PINK1 result in decreased complex I activity and deficient synaptic function. *EMBO Mol. Med.* ***2009***, 1, 99 –111.
31. Sire´n, A.; Polvi, A.; Chahine, L.; Labuda, M.; Bourgoin, S.; Anttonen, A.K.; Kousi, M.; Hirvonen, K.; Simola, K.O.; Andermann, E.; et al. Suggestive evidence for a new locus for epilepsy with heterogeneous phenotypes on chromosome 17q. *Epilepsy Res.* **2010**, 88, 65–75.
32. Babenko, V.M.; Smagin, D.M.; Galyamina, A.G.; Kovalenko, I.L.; Kudryavtseva,

N.N. Altered Slc25 Family Gene Expression as Markers of Mitochondrial Dysfunction in Brain Regions Under Experimental Mixed Anxiety/Depression-Like Disorder. *BMC Neurosci.* **2018**, 19, 79.

33. Norheim, K.B.; Hellard, S.L.; Nordmark, G.; Harbose, E.; Gøransson, L.; Brun, J.G.; Wahern-Herlenius, M.; Jonsson, R.; Omdal, R. A Possible Genetic Association With Chronic Fatigue in Primary Sjögren's Syndrome: A Candidate Gene Study. *Rheumatol. Int.* **2014**, 34, 191–197.

34. Strawbridge, R.J.; Ward, J; Lyall, L.M.; Tunbridge, E.M.; Cullen, B.; Graham, N.; Ferguson, A.; Johnston, K.J.A.; Lyall, D.M.; Mackay, D.; et al. Genetics of self-reported risk taking behaivior, trans-ethnic consistency and relevance to brain gene expression. *Transl Psychiatry* **2018,** 8, 178.

35. Price, J.B.; Bronars, C.; Erhardt. S.; Cullen, K.R.; Schwieler, L.; Bert, M.; Walder, K.; McGee, S.L.; Frye, M.; Tye, S.J. Boenergetics and synaptic plasticity as potential targets for individualizing treatment for depression. *Neurosci. Biobehav. Rev.* **2018**, 90, 212-220.

36. Laguesse, S.; Morisot, N.; Shin, J.H.; Liu, F.; Adrover, M.F.; Sakhai, S.A.; Lopex, M.F.; Phamluong, K.; Griffin 3rd, W.C.; Becker, H.C.; et al. Prosapip1-Dependent Synaptic Adaptations in the Nucleus Accumbens Drive Alcohol Intake, Seeking, and Reward. *Neuron* **2017**, 96, 145-159.

37. Schneeberber, P.E.; Bierhals. T.; Neu, A.; Hempel, M.; Kutsche, K. de novo MEOCE nonsense variant associated with a neurodevelopmental disorder causes disintegration of 7SK snRNP and enhance d RNA polymerase II activation. *Sci. Rep.* **2019**, 9, 12516

38. Broder, C.; Becker-Pauly, C. The metalloproteases meprin alpha and meprin beta: unique enzymes in inflammation, neurodegeneration, cancer and fibrosis. *Biochem. J.* **2013**, 450: 253-264.

39. Banerjee, S.; Oneda, B.; Yap, L.M.; Jewell, D.P.; Matters, G.L.; Fitzpatrick, L.R.; Seibold, F.; Sterchi, T.; Ahmad, T.; Lottaz, D.; Bond, J.S. MEP1A allele for meprin A metalloprotease is a susceptibility gene for inflammatory bowel disease. *Mucosal Immunol.* **2009,** 2: 220- 231.

40. Lam, U.D.P.; Lerchbaum, E.; Schweighofer, N.; Trummer, O.; Eberhard, K.; Genser, B.; Piever, T.R.; Obermayer-Pietsch, B. Association of MEP1A gene variants with insulin metabolism in central European women with polycystic ovary syndrome. *Gene* **2014**, 2014, 245-23

41. Abbracchio, M.P., Burnstock, G., Verkhratsky, A., Zimmermann, H. Purinergic signalling in the nervous system: an overview. *Trends Neurosci.* **2009**, 32, 19–29.

42. Parvathenani, L.K.; Tertyshnikova, S.; Greco, C.R.; Roberts, S.B.; Robertson, B.; Posmantur, R. P2X7 Mediates Superoxide Production in Primary Microglia and Is Up-regulated in a Transgenic Mouse Model of Alzheimer's Disease. *J Biol Chem.* **2003,** 278, 13309–13317.

43. Ali-Sisto, T.; Tolmunen, T.; Toffol, E.; Viinamäki, H.; Mäntyselkä, P.; Valkonen-Korhonen, M.; Honkalampi, K.; Ruusunen, A.; Velagapudi, V.; Lehto, S.M. Purine metabolism is dysregulated in patients with major depressive disorder. *Psychoneuroendocrinology* **2016**, 70, 25-32.

44. Yugel, M.E.; Shah, K.D.; Brown, E.B.; Burns, C.; Bennick, R.A.; diangelo, J.R.; Keene, A.C. Ade 2 functions in the drosophila fat body to promote sleep. *G3(Bethesda)* **2018**, 8, 3385-3395.

45. Lee, J.S.; Yoo, Y.; Lim, B.C.; Kim, K.J.; Song, J.; Choi, M.; Chae, J.H. GM3 synthase deficiency due to **ST3GAL5** variants in two Korean female siblings: Masquerading as Rett syndrome-like phenotype. *Am. J. Med. Genet. A.* **2016,** 170, 2200-2205

46. Gordon-Lipkin, E.; Cohen, J.S.; Srivastaca, S.; Soares, B.P.; Levey, E.; Fatemi, A. ST3GAL5-Related Disorders: A Deficiency in Ganglioside Metabolism and a Genetic Cause of Intellectual Disability and Choreoathetosis. *J. Child. Neurol.* **2018**, 33, 825-831.

47. Zandi, P.P.; Avramopoulos, D.; Willour, V.L.; Huo, Y.; Miao, K.; Mackinnon, D.F.; McInnis, M.G.; Potash, J.B.; Depaulo, J.R. SNP fine mapping of chromosome 8q24 in bipolar disorder. *Am. J. Med. Genet. B Neuropsychiatr. Genet.* **2007**, 144B, 625–630.

48. Perlis, R.H.; Pucell, S.; Fagerness, J.; Kirby, A.; Petryshen, T.L.; Fan, J.; Sklar, P. Family-Based Association Study of Lithium-Related and Other Candidate Genes in Bipolar Disorder. *Arch. Gen. Psychiatry* **2008**, 65(1), 53-61

49. Zhang, P.; Xiang, N.; Chen, Y.; Œliwerska E.; McInnise, M.G.; Burmeister, M.; Zöllner, S. Family-based association analysis to finemap bipolar linkage peak on chromosome 8q24 using 2,500 genotyped SNPs and 15,000 imputed SNPs. *Bipolar* Disord. **2010**, 12, 786–792.

Yoshida, K.; Higuchi, H.; Takahashi, H.; Kamata, M.; Sato, K.; Inoue, K.; Suzuki, T.;

Itoh, K.; Ozaki, N. (2008). Influence of the tyrosine hydroxylase val81met polymorphism and catechol-O-methyltransferase val158met polymorphism on the antidepressant effect of milnacipran. *Hum. Psychopharmacol.* **2008,** 23, 121–128.
